# Supplementary material for: Silencing cortical activity during sound-localization training impairs auditory perceptual learning
Source: Nat Commun. 2019 Jul 12;10:3075. doi: 10.1038/s41467-019-10770-4 (PMC6625986; doi:10.1038/s41467-019-10770-4)
Supplement: Supplementary file 1 — Supplementary Information [file 41467_2019_10770_MOESM1_ESM.pdf]

Silencing cortical activity during sound-localization training impairs auditory perceptual learning

Bajo et al.

Supplementary Figures 1 to 6

Supplementary Table 1

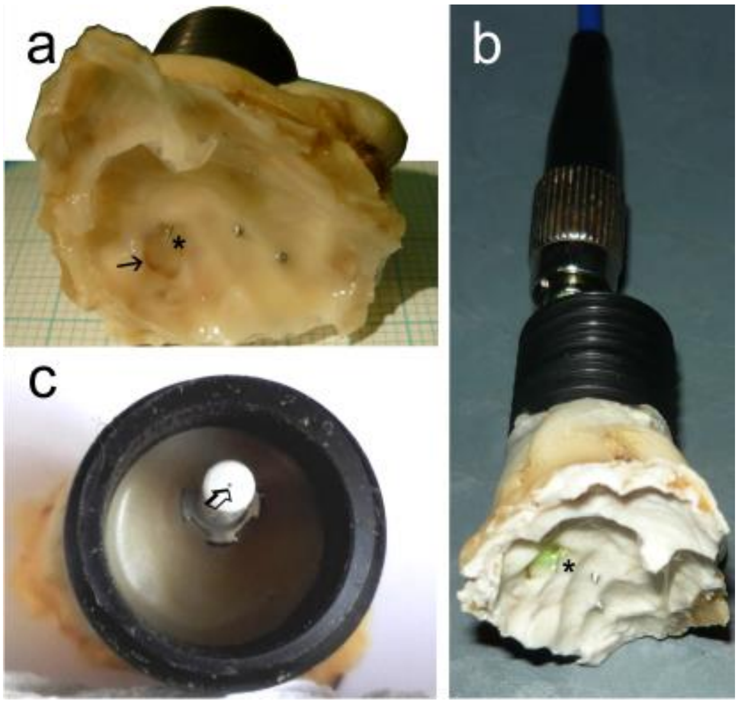

Supplementary Figure 1: Optical permeability of the fiber-optic cannulae 30 months post-implantation.

Once the behavioral testing had been completed and the animals perfused for histological confirmation of transfection levels, each chronic implant containing a fiber-optic cannula was dissected along with the temporal part of the skull to ensure that there was no bone growth over the end of the cannula. After inspection under a microscope, the optical fiber was reconnected to the laser source to confirm that the beam was not obstructed and the light intensity remained unchanged. (**a**) Inner view at the level of the temporal skull after the brain had been removed, showing the tips of the two screws used to anchor the implant, the craniotomy (arrow) where the ectosylvian sulcus was located and the fiber-optic cannula at the top right corner of the craniotomy (asterisk). (**b**) Different view of the same implant connected to the laser to illustrate the permeability of the optical fiber (asterisk, green light can be seen beneath the skull). (**c**) Dorsal view of the external part of the same implant, which included a protective circular enclosure, within which the zirconia ferrule of the optical fiber can be observed (open arrow). Pictures were taken from animal F1111 and are representative of every ferret implanted (n = 15, Supplementary Table 1, asterisks).

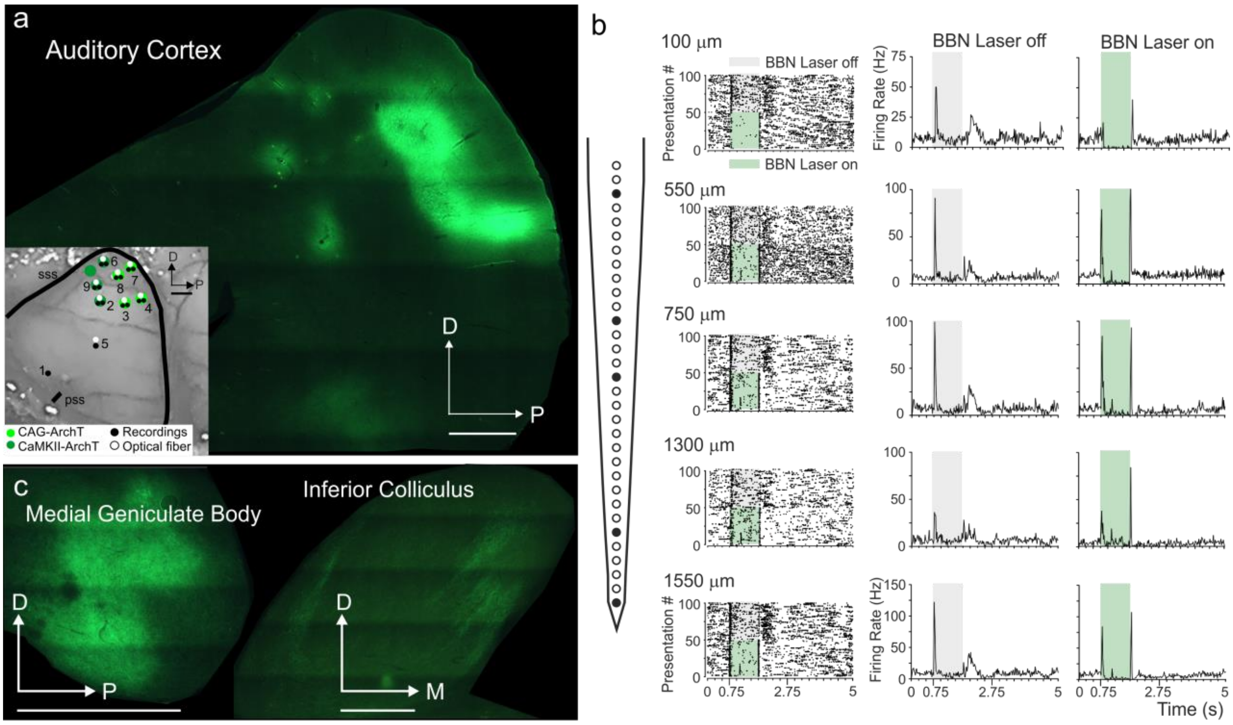

Supplementary Figure 2: Optogenetic suppression of sound-driven activity in A1.

(**a**) Histological section of a flattened left auditory cortex (ferret F1804) showing GFP immunofluorescence associated with ArchT expression using two different promoters. The inset shows a photograph of the cortical surface of this animal on which the locations of the recording sites and optical fiber placements in the dorsal high-frequency region of A1 have been marked. (**b**) Examples of unit responses recorded at location #7 (AAV8/CAG-ArchT-GFP injection) at 5 different cortical depths, which are indicated by the solid black circles on the schematic of the recording probe (distance between adjacent recording sites, 50 µm). For each unit, dot rasters show the responses to 100 presentations of broadband noise (BBN, 1000 ms duration), grouped into laser-off (top half, gray) and laser-on presentations (bottom half, green) in the same fashion as during behavioral testing. Each sweep lasted 5 s (comprising 750 ms of spontaneous activity preceding the 1,000-ms stimuli followed by 3,250 ms after laser offset) so that the time-course of optogenetic suppression could be measured. The next two columns show the corresponding peristimulus time histograms (20 ms bins) for BBN only (left) and BBN plus laser illumination (right). All 5 units exhibited a significant suppression of acoustically-driven activity (comparison of activity during a time window from 760-1,760 ms for BBN laser-off vs BBN laser-on presentation, t-test, $P < 0.05$ for each unit). During sound presentation, the firing rate of these example units was reduced by laser illumination to different extents (85%-25%), but in each case the prominent offset response observed in the absence of laser illumination was eliminated when the laser was turned on (comparison of pre-stimulus spontaneous activity vs offset response during a time window from 1,800-2,000 ms; laser-off, t-test, $P < 0.05$; laser-on, $P > 0.165$). The activity of all the units recovered soon after the laser was turned off (comparison of pre-stimulus spontaneous activity vs activity 0.5 s after the laser was turned off, $P > 0.502$ for each unit). (**c**) Histological sections at the level of the ipsilateral medial geniculate body and inferior colliculus showing the terminal fields of the axons of descending cortical projection neurons expressing GFP. Calibration bars in **a** and **c**, 1 mm. D, dorsal; M, medial; P, posterior; pss, pseudosylvian sulcus; sss, suprasylvian sulcus.

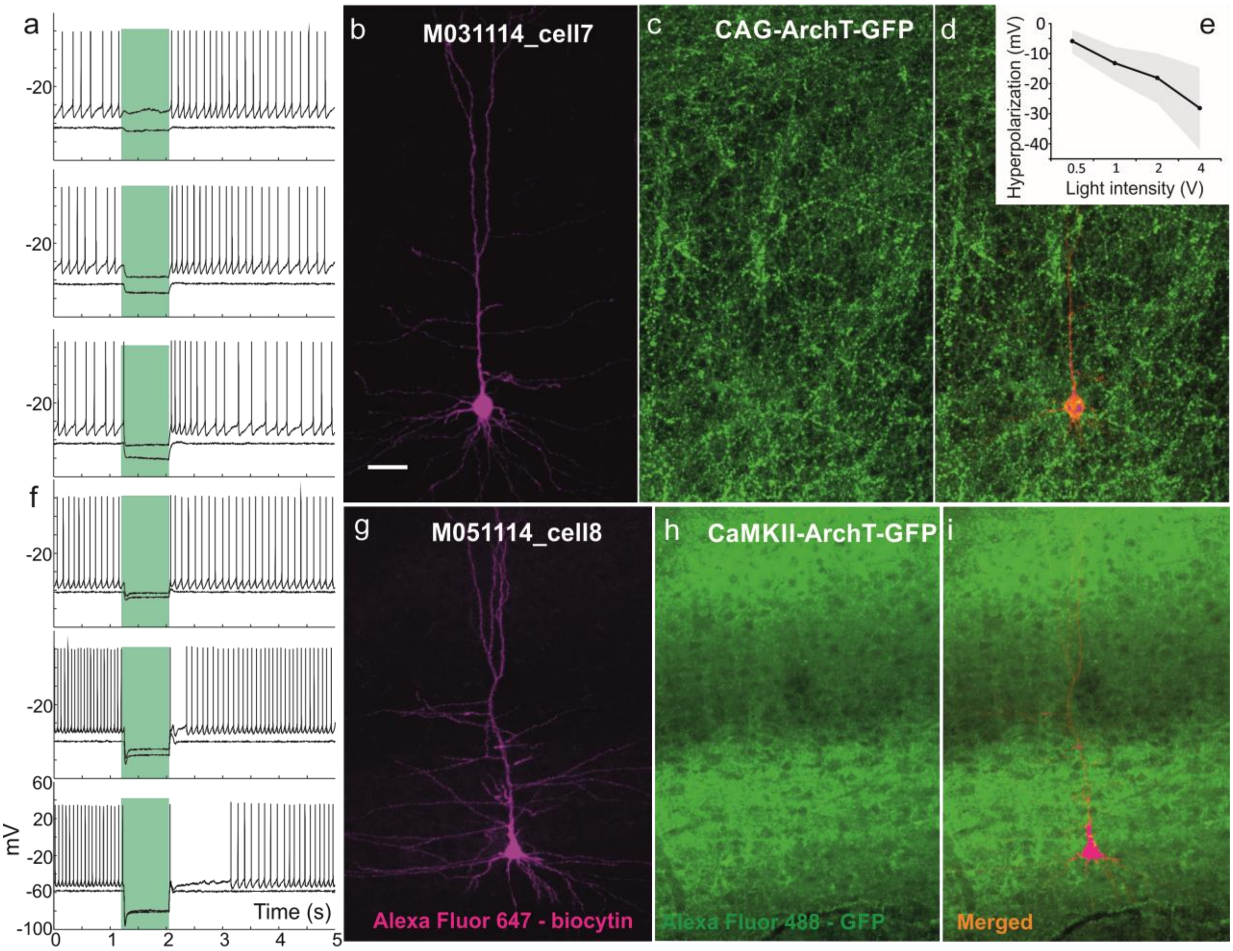

Supplementary Figure 3: *In vitro* whole-cell recordings to validate the ArchT constructs.

(**a**) Effect of laser light pulses (green rectangles) of different magnitudes on the activity of a pyramidal neuron in mouse motor cortex. This neuron was filled with biocytin for identification (**b**) and expressed ArchT-GFP under the CAG promotor (**c**). (**d**) Merged image (colocalization of both chromophores is shown in yellow/orange). (**e**) Linear relationship (R^2 = 0.982) between the magnitude of the hyperpolarizing response and the intensity of the light pulse. The black line indicates the mean, with the standard deviation in gray (n = 6 cells). (**f**) Effect of light pulses (green rectangles) of different magnitudes on the activity of a second pyramidal neuron in mouse motor cortex. This neuron was filled with biocytin (**g**) and expressed ArchT-GFP under the CaMKII promotor (**h**). (**i**) Merged image (colocalization of both chromophores is shown in yellow/orange). Calibration bar in **b** represents 100 μm and applies to **c**, **d** and **g-i.** The light pulses hyperpolarized the cells and completely suppressed action potential firing regardless of the promoter used (hyperpolarization/LEDv = 10.7 ± 6 mV/V). Although the resting membrane potential recovered rapidly (<0.5 s) after laser offset, spiking activity (measured by the duration of the interspike intervals) took longer to return to baseline levels. When the CAG promoter was used to target every cell type at the injection site, the hyperpolarization and cessation of firing caused by the laser pulse were normally followed by a single rebound period of higher firing, which then returned to pre-laser activity levels within 1-3 s after laser offset (**a**). The firing pattern observed post-illumination was usually more complex when excitatory pyramidal neurons were targeted by expressing ArchT under the CaMKII promoter, which included a combination of rebound firing and a further period of silence, and took longer to return to the pre-light activity. The duration of the post-light suppression of firing (1-4 s) depended on light intensity (**f**). This difference cannot be attributed to differences in the type of neurons from which these recordings were obtained, which all had a pyramidal morphology, as shown by filling them with biocytin, or to variation in ArchT expression levels, but more likely reflects the overall effect of illumination on the cortical networks in which different populations of neurons expressed ArchT according to the promotor used.

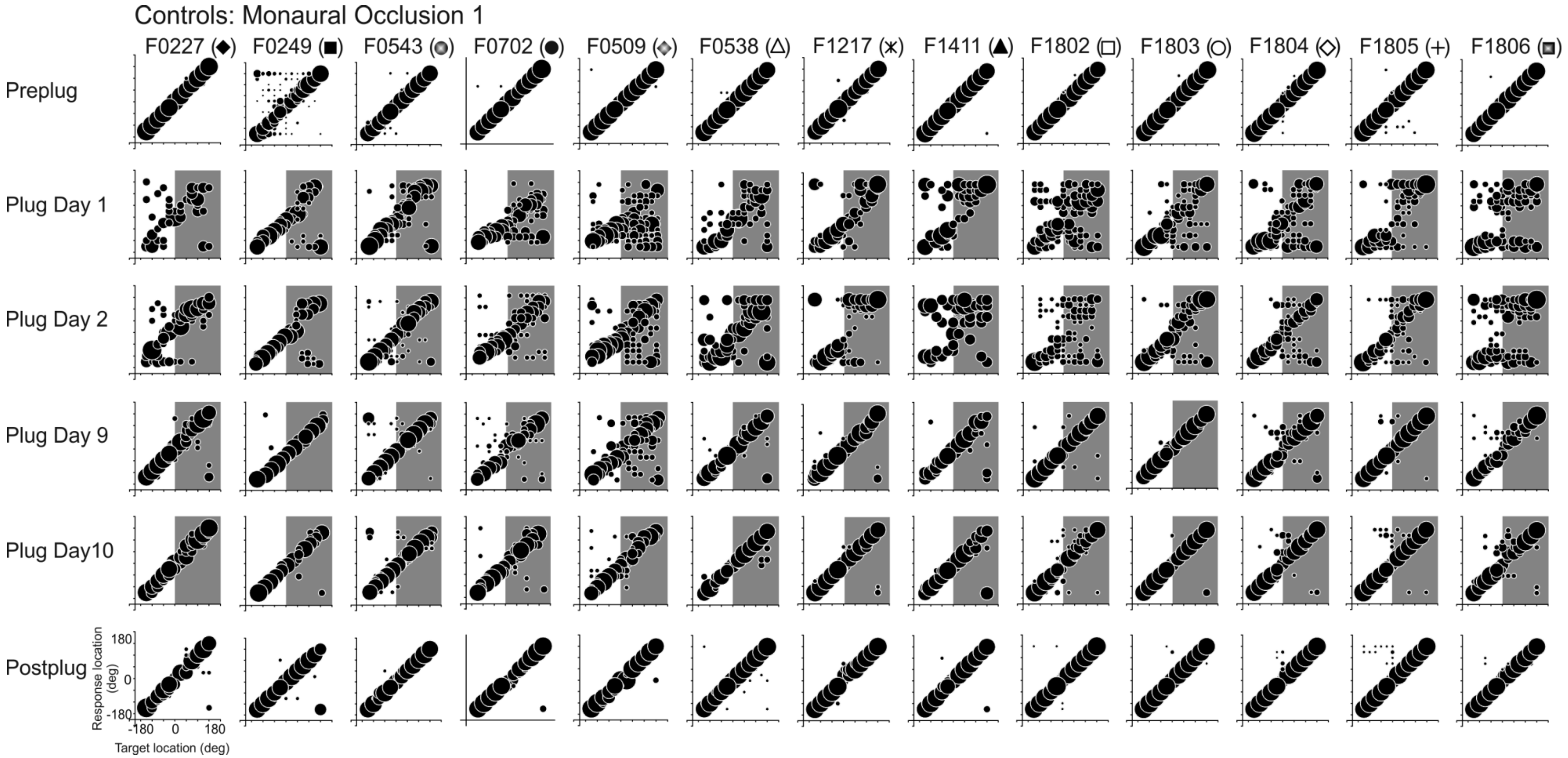

Supplementary Figure 4: Effects of monaural occlusion on auditory localization in control ferrets.

Stimulus-response plots showing the performance of each control animal before (Preplug), during (Plug Day 1, 2, 9, 10) and after (Postplug) the first period of monaural occlusion. Each column shows data from an individual animal, with the ferret number at the top of the column (see Supplementary Table S1). Symbols identify these animals in Figures 4 and 6. The first and final rows show data from the two days before and after monaural occlusion, respectively. The middle four rows show data from the first two and last two days of monaural occlusion, respectively. Negative and positive angles indicate sound locations in the left and right hemifields, respectively. The gray rectangle indicates that the right ear was plugged.

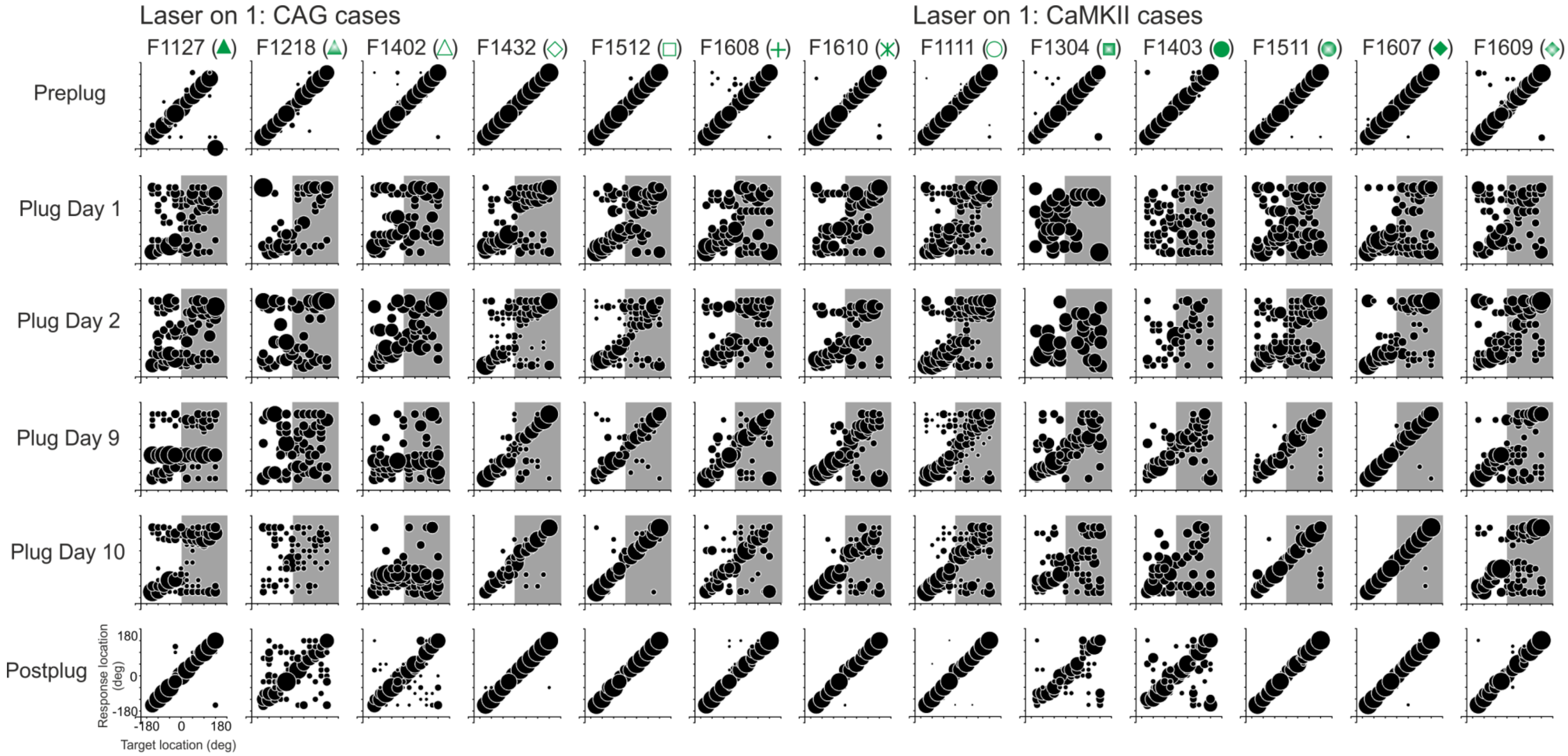

Supplementary Figure 5: Effects of monaural occlusion on auditory localization in ArchT ferrets.

Stimulus-response plots showing the performance of each 'ArchT-laser on' animal before (Preplug), during (Plug Day 1, 2, 9, 10) and after (Postplug) the first period of monaural occlusion. Each column shows data from an individual animal, with the ferret number at the top of the column. Symbols identify these animals in Figures 4 and 6. In the first seven cases, the CAG promoter was used in the viral construct (AAV8/CAG-ArchT-GFP) and in the last six cases the CaMKII promoter was used (AAV8/CaMKII-ArchT-GFP; See Supplementary Table 1). The first and final rows show data from the two days before and after monaural occlusion, respectively. The middle four rows show data from the first two and last two days of monaural occlusion, respectively. Negative and positive angles indicate sound locations in the left and right hemifields, respectively. The gray rectangle indicates that the right ear was plugged.

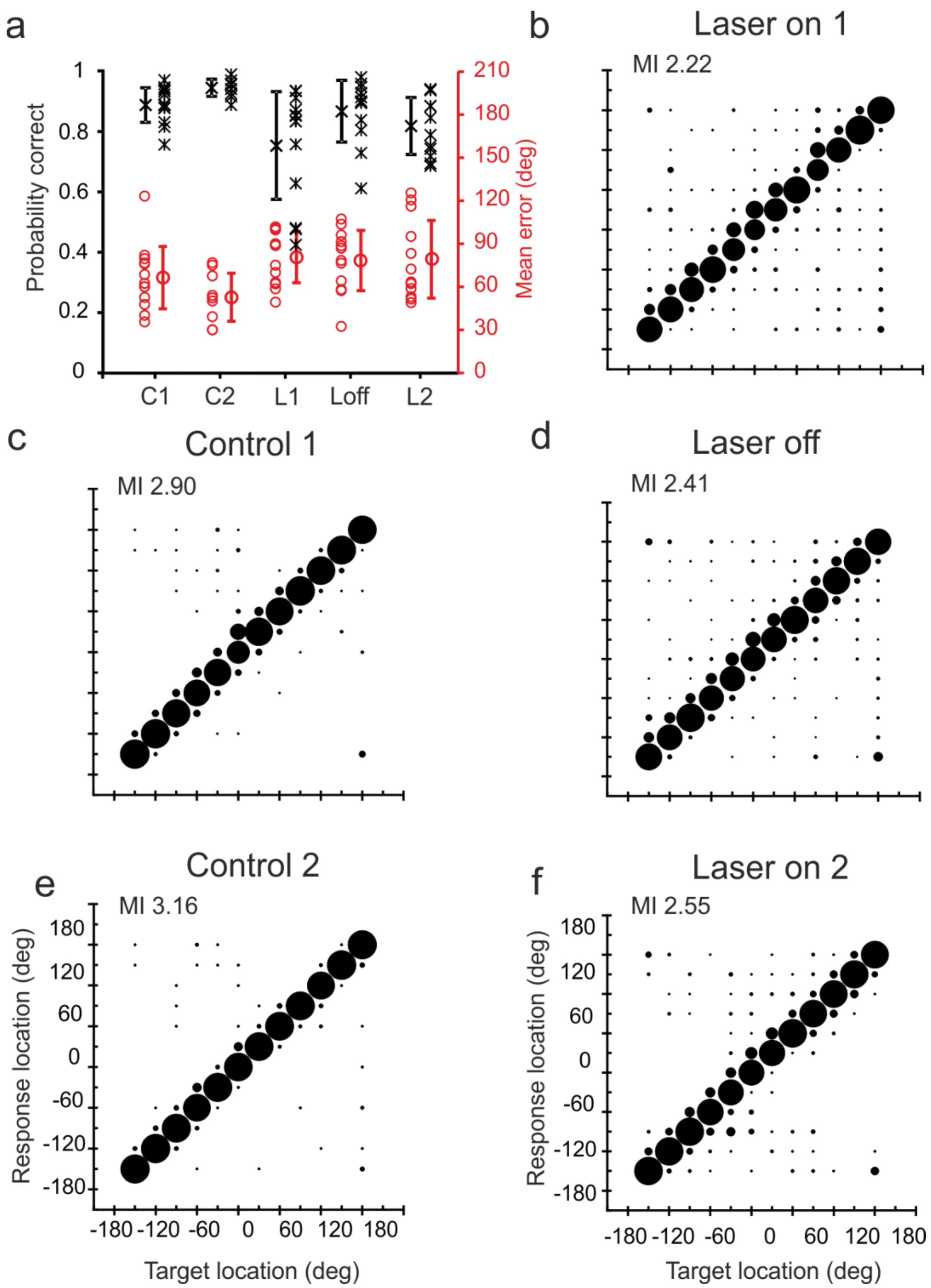

Supplementary Figure 6: Performance after removal of the earplug.

(**a**) Probability of correct responses (asterisks) and mean error magnitude on incorrect trials (open circles) for the different groups (C1and C2, control groups during first and second period of monaural occlusion; L1, Loff and L2 ArchT groups during the different periods of monaural occlusion and laser activation). Each symbol represents a different animal and symbols with error bars correspond to mean values. (**b**-**e**) Stimulus-response plots based on all the trials across all animals in each group. In each panel, the mutual information (MI in bits) between target and response locations is included. A value of 3.58 bits corresponds to perfect performance. The performance of the control animals was more accurate and less variable than that of the ferrets in which ArchT was expressed in A1, indicating a delayed return to normal localization following monaural occlusion. Sample sizes: n = 26 (**a**), n = 13 (**b**, **d**), n = 12 (**f**), n = 13 (**c**), n = 9 (**e**).

Supplementary Table 1: List of animals used in this study.

Animal identity	Behavior	Recordings	Anatomy	Promoter/control	Figures
Ferret 1127*	SL & 3 MO		Fl + ABC	CAG	Figs. 1c-e, 3 Quant., 4, 5, 6b-c, S5 & S6a, b, d, f.
Ferret 1218*§	SL & 2 MO		Fl + ABC	CAG	Figs. 1c-e, 3 Quant., 4, 5, 6b-c, S5 & S6a, b, d.
Ferret 1402*	SL & 3 MO		Fl	CAG	Figs. 1c-e, 4, 5, 6b-c, S5 & S6a, b, d, f.
Ferret 1432*	SL & 3 MO	4 shanks	Fl	CAG	Figs. 1c-e, 4, 5, 6b-c, S5 & S6ab, d, f.
Ferret 1512*	SL & 3 MO	4 shanks	Fl	CAG	Figs. 1c-e, 4, 5, 6b-c, S5 & S6a, b, d, f.
Ferret 1608*	SL & 3 MO	4 shanks	Fl	CAG	Figs. 1c-e, 4, 5, 6b-c, S5 & S6a, b, d, f.
Ferret 1610*	SL & 3 MO	4 shanks	Fl	CAG	Figs. 1c-e, 4, 5, 6b-c, S5 & S6a, b, d, f.
Ferret 1111*	SL & 3 MO		Fl + ABC	CaMKII	Figs. 1c-e, 3 Quant., 4, 5, 6b-c, S1, S5 & S6a, b, d, f.
Ferret 1304*	SL & 3 MO		Fl	CaMKII	Figs. 1c-e, 4, 5, 6b-c, S5 & S6a, b, d, f.
Ferret 1403*	SL & 3 MO		Fl	CaMKII	Figs. 1c-e, 4, 5, 6b-c, S5 & S6a, b, d, f.
Ferret 1511*	SL & 3 MO		Fl	CaMKII	Figs. 1c-e, 4, 5, 6b-c, S5 & S6a, b, d, f
Ferret 1607*	SL & 3 MO		Fl	CaMKII	Figs. 1c-e, 4, 5, 6b-c, S5 & S6a, b, d, f.
Ferret 1609*	SL & 3 MO		Fl	CaMKII	Figs. 1c-e, 4, 5, 6b-c, S5 & S6a, b, d, f.
Ferret 1115		1 shank	Fl + ABC	CAG	Fig. 3 & Quant.
Ferret 1431		1 shank	Fl	CaMKII	Fig. 2a-d.
Ferret 1003			Fl	CaMKII	
Ferret 1110			Fl + ABC	CaMKII	Fig. 3 Quant.
Ferret 1120			Fl	CAG	
Ferret 1804	SL & 2 MO	2 shanks	Fl	Control (earplug)	Figs. 2e-h, 4b-d, 5, 6a, S2, S4 & S6a, c, e.
Ferret 0227§	SL & 1 MO			Control (earplug)	Fig. 4b-c, 5, 6a, S4 & S6a, c.
Ferret 0249§	SL & 1 MO			Control (earplug)	Fig. 4b-c, 5, 6a, S4 & S6a, c.
Ferret 0543§	SL & 1 MO			Control (earplug)	Fig. 4b-c, 5, 6a, S4 & S6a, c.
Ferret 0702§	SL & 1 MO			Control (earplug)	Fig. 4b-c, 5, 6a, S4 & S6a,c.
Ferret 0509	SL & 2 MO			Control (earplug)	Figs. 4b-d, 5, 6a, S4 & S6a, c, e.
Ferret 0538	SL & 2 MO			Control (earplug)	Figs. 4b-d, 5, 6a, S4 & S6a, c, e.

Ferret 1217*	SL & 2 MO		Fl + ABC	Control (light)	Figs. 4b-d, 5, 6a, S4 & S6a, c, e.
Ferret 1411*	SL & 2 MO		Fl	Control (no ArchT)	Figs. 4b-d, 5, 6a, S4 & S6a, c, e.
Ferret 1802	SL & 2 MO			Control (earplug)	Figs. 4b-d, 5, 6a, S4 & S6a, c, e.
Ferret 1803	SL & 2 MO			Control (earplug)	Figs. 4b-d, 5, 6a, S4 & S6a, c, e.
Ferret 1805	SL & 2 MO			Control (earplug)	Figs. 4b-d, 5, 6a, S4 & S6a, c, e.
Ferret 1806	SL & 2 MO			Control (earplug)	Figs. 4b-d, 5, 6a, S4 & S6a, c, e.
Mouse M031114		*In vitro*	Fl	CAG	Fig. S3a-e.
Mouse M051114		*In vitro*	Fl	CaMKII	Fig. S3e-i.
Mouse M010882			Fl	CAG	
Mouse M029884			Fl	CaMKII	
Mouse M049881			Fl	CaMKII	

Thirty-one ferrets comprising 18 experimental animals (blue) and 13 controls (black), and five mice were used. Different colors in the table cells indicate different procedures: behavior (green), electrophysiological recordings (orange), and anatomy (yellow). An asterisk after the animal name indicates that it was implanted and § indicates when the full set of behavior was not completed. The last column shows which animals contribute data to specific figure panels. Gaps in the table separate animals used for different combinations of procedures: behavior with ArchT using GAG promoter; behavior with ArchT using CaMKII promoter; electrophysiological recording with ArchT; anatomy with ArchT; ferret controls; *in vitro* mouse recordings with ArchT; *in vitro* mouse anatomy with ArchT. One of the control behavioral animals (F1804) was later used to record neural activity after ArchT was expressed in the auditory cortex (see Supplementary Fig. 2). It was the only case where both constructs, AAV8/CAG-ArchT-GFP and AAV8/CaMKII-ArchT-GFP, were injected in the same animal, at specific locations in the primary auditory cortex.

Viral construct injections were made in every animal where Fl (fluorescence) is indicated in the Anatomy column. In each case, the location of the injection sites in the primary auditory cortex and the presence of GFP-labeled axons and terminals in the contralateral cortex, ipsilateral MGB and bilaterally in the IC was examined using confocal microscopy. ABC in the anatomy column indicates that one set of sections was used for permanent labeling of NeuN- and GFP-positive cells with the avidin biotin complex system; those cases were used for stereological quantification. F1217 was excluded from the quantification due to the absence of GFP-positive neurons, indicating no viral transfection; this animal was therefore treated as a control for the effect of the green light delivery to the auditory cortex during training.

Abbreviations: ABC, avidin biotin complex; CAG, synthetic promoter containing cytomegalovirus early enhancer, the 1st exon and intron of chicken beta-actin gene as promoter, and the splice acceptor of the rabbit beta-globin gene; CamKII, Ca^{2+} calmodulin-dependent protein kinase II; Fl, fluorescence; MO, 10-day periods of monaural occlusion; SL, sound localization behavior.
